# Emergence of Dengue Virus Serotypes 1 and 3 in Mahottari and Adjacent Areas of Southern Nepal

**DOI:** 10.3390/pathogens14070639

**Published:** 2025-06-26

**Authors:** Sabin Shrestha, Sandesh Rimal, Anjana Kharbuja, Manoj Kumar Ray, Susmita Shrestha, Anjali Dulal, Suprabha Subedi, Ashma Khadka, Nabaraj Adhikari, Meghnath Dhimal, Basu Dev Pandey, Takeshi Urano, Kouichi Morita, Mya Myat Ngwe Tun, Shyam Prakash Dumre

**Affiliations:** 1 Central Department of Microbiology, Tribhuvan University, Kathmandu 44601, Nepal; sabeinartha@gmail.com (S.S.); niensandesh@gmail.com (S.R.); kharbujaanjana@gmail.com (A.K.); anjalidulal1998@gmail.com (A.D.); khadka2aashma@gmail.com (A.K.); adhikarinaba13@gmail.com (N.A.); 2 Bardibas Hospital, Mahottari 45700, Nepal; raymanojtechnologist@gmail.com; 3 Share and Care Medical Center Private Limited, Sunsari 56700, Nepal; susmitashrestha741@gmail.com; 4 Nepal Health Research Council, Ram Shah Path, Kathmandu 44600, Nepal; meghdhimal@gmail.com; 5 DEJIMA Infectious Diseases Research Alliance, Nagasaki University, Nagasaki 852-8523, Japan; drbasupandey@gmail.com (B.D.P.); moritak@nagasaki-u.ac.jp (K.M.); 6 Center for Vaccines and Therapeutic Antibodies for Emerging Infectious Diseases, Shimane University, Izumo 690-8504, Japan; turano@med.shimane-u.ac.jp; 7 Department of Tropical Viral Vaccine Development, Institute of Tropical Medicine, Nagasaki University, Nagasaki 852-8523, Japan

**Keywords:** dengue, DENV serotype, viral load, epidemiology, outbreak, surveillance, Nepal

## Abstract

Dengue has been a serious public health concern in Nepal since the past few years, with concurrent big outbreaks occurring in 2022–2024. This cross-sectional study was conducted among febrile patients visiting hospitals in Mahottari district in southern Nepal. A total of 2141 dengue-suspected patients were investigated by routine laboratory assays and serological and molecular techniques, including real-time quantitative polymerase chain reaction (RT-qPCR). Among them, 455 (21.3%) were confirmed as dengue cases. The majority of dengue cases (435, 95.6%) had a primary dengue infection. The total bilirubin level was significantly higher in secondary dengue infection than in primary (*p* = 0.032). The major dengue virus (DENV) serotypes responsible for this outbreak were DENV-1 (45.5%) and DENV-2 (40.9%), while 13.6% patients had DENV-3 infection. DENV-3 infection showed a significantly higher viral load (median: 7.71 Log_10_ copies/mL; range: 6.48–7.94) compared to DENV-1 (6.72 Log_10_ copies/mL; 5.49–7.17) and DENV-2 (4.76 Log_10_ copies/mL; 2.32–6.96). Adult patients exhibited a significantly higher viral load than children (*p* = 0.035). NS1- and IgM-positive as well as admitted patients had a higher viral load (*p* < 0.05). Co-circulation of multiple serotypes (DENV-1, -2, -3) was confirmed with the first introduction of DENV-1 and DENV-3 in Mahottari and surrounding areas in the 2023 outbreak. Identification of the circulating DENV serotypes is crucial to understanding the epidemiological trend and dynamics of population immunity. These findings underscore the need of nation-wide integrated surveillance, including genomic data generation, in Nepal for disease control, prevention, and potential vaccine implication.

## 1. Introduction

Dengue is a mosquito-borne viral infection caused by any of the four closely related dengue virus (DENV) serotypes (DENV-1 to -4) and transmitted by female *Aedes* mosquitos (*A. aegypti* and *A. albopictus*) in the human population [1,2,3]. DENV isan enveloped virus belonging to the family Flaviviridae and genus Orthoflavivirus with a single-stranded RNA genome that encodes three structural (capsid, membrane, and envelope) and seven non-structural proteins (NS1-NS5) [4,5,6]. Clinically, DENV infection ranges from asymptomatic to dengue with or without warning signs or severe dengue [7]. Dengue is mainly prevalent in tropical and subtropical regions, including parts of Asia, the Americas, Africa, the Pacific Islands, and northern Australia. The disease is endemic in 129 countries, with one-third of the global population at risk of infection [1]. The Southeast Asia (SEA) region is a hotspot for DENV, comprising over 50% of global cases. In 2023 alone, there were more than 6 million dengue cases, with more than 6000 dengue-related deaths reported from 92 countries [2,3,8]. In 2024, dengue cases surpassed 14 million, with over 10,000 dengue-related deaths globally [9]. A substantial proportion of DENV infections remain undetected or underreported due to their asymptomatic or mild nature. Of the estimated 105 million DENV infections occurring annually worldwide, only approximately 51 million manifest with clinical symptoms. [10]. The global prevalence of asymptomatic DENV infections is estimated at 59.3%, with higher rates observed during outbreaks (65.5%) compared to non-outbreak periods (30.8%) [11]. Among symptomatic cases, only a minority progress to severe dengue, while the majority present with warning signs but do not develop severe disease [7,12].

In Nepal, the first dengue infection was reported in a Japanese volunteer working in the southern part of the country in 2004 [13]. The first endogenous outbreak was recorded in 2006, after which Nepal experienced major dengue outbreaks in 2010, 2013, 2016, 2019, 2022, and 2023 [14,15,16,17,18]. Despite this, fatalities due to dengue remained very low in Nepal. However, following the 2022 dengue epidemic, with over 54,000 cases and 88 deaths, another unprecedented outbreak unfolded in 2023, recording over 51,000 cases and 20 deaths in Nepal [14,19]. Additionally, in 2024, 34,385 dengue cases, along with 13 fatalities, were reported [20].

Bardibas municipality, located in Mahottari district of southern Nepal (the study site), reported its first outbreak of dengue in 2017, with 847 cases recorded in a two-week period [21]. However, according to the 2017 data from the Epidemiology and Disease Control Division (EDCD) of Nepal, the total number of reported dengue cases in Mahottari district was 438. In subsequent years, Mahottari recorded 3 cases in 2018, 6 cases in 2019, 61 cases in 2022, and 37 cases in 2023 [19,22]. This national dengue data seems to be largely underreported for potential dengue hot spots like Mahottari. Major factors responsible for underreporting in the resource limited settings are a lack of diagnostic facilities (both limited access and availability), poor or non-compliance to reporting mechanisms, and a lack of an integrated surveillance system in Nepal [23].

Mahottari and neighboring districts have open borders with the dengue-endemic state of India and a massive connectivity with other parts of Nepal through national highways, which make these regions more prone to dengue and other mosquito-borne diseases through cross-border as well as intra-country transmission. However, the precise epidemiology of dengue and its serotype distribution in this area remains largely unexplored. Therefore, we report a comparative laboratory profile analysis serotype distribution, and viral load perspectives of the 2023 dengue outbreak in Mahottari district and adjacent areas of southern Nepal.

## 2. Materials and Methods

### 2.1. Study Design and Sites

**Figure 1:**
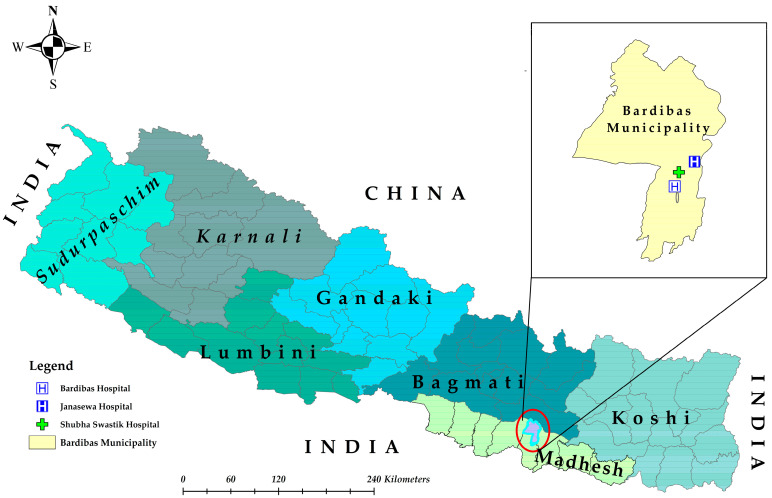
Map of Nepal showing the study area (Bardibas municipality, Mahottari district, Madhesh province).

This prospective cross-sectional study was conducted in Mahottari district of southern Nepal, which borders Dhanusha district in the east, Sarlahi in the west, Sindhuli in the north, and India in the south (Figure 1). This district extends from 26°38′0″ N latitude to 85°48′0″ E longitude and covers an area of 1002 km^2^, and it is home to 705,838 people. The elevation ranges from 136 m above sea level (ASL) to 774 m ASL [24]. Mahottari has a humid, subtropical, and dry winter climate. The yearly average temperature is 29.1 °C (22.32–32.52 °C), which is 7.1 °C higher than Nepal’s average temperature. It typically receives about 139.49 mm of precipitation and has 134.3 rainy days (36.8% of the time) annually [25].

The study hospitals (Bardibas, Shubha Swastik and Janasewa) are situated in the Bardibas municipality of Mahottari. Among them, Bardibas hospital is a public hospital, while the other two hospitals are privately owned. All these hospitals are equipped for testing and management of dengue-suspected patients. These hospitals primarily serve patients from Mahottari, Dhanusha, and Sarlahi districts as well as neighboring districts such as Sindhuli, Siraha, Rautahat, Sunsari, and others.

### 2.2. Patient Enrollment, Questionnaire, and Sample Collection

Dengue-suspected febrile patients with any two of the following symptoms: fever, nausea, vomiting, rash, aches, and pains, visiting the study hospitals were included in the study regardless of their age or gender or original locations. A standardized questionnaire was filled out by interviewing the patients or their caretakers to gather information on patients’ demographic details. A blood sample (3–5 mL) was collected by venipuncture, and one part was utilized for routine laboratory analysis. Serum was separated from the second portion, and a rapid diagnostic test (RDT) for dengue was employed. The remaining serum was aliquoted to three vials and transported to and stored at −80 °C in the laboratory of Central Department of Microbiology (CDMi), Tribhuvan University, Kathmandu, Nepal, until further analysis.

### 2.3. Dengue Diagnosis

Dengue infection was diagnosed by rapid diagnostic kits using Bioline™ DENGUE DUO (DENGUE NS1 Ag + IgG/IgM) (Abbott Diagnostics, Korea Inc., Gyeonggi-do, Republic of Korea) and/or conventional RT-PCR ((Takara Bio Inc., Shiga, Japan) as per the kit manufacturers’ instructions. Further, they were also categorized based on immune status (primary or secondary infection) and admission status (inpatients and outpatients).

**Figure 2:**
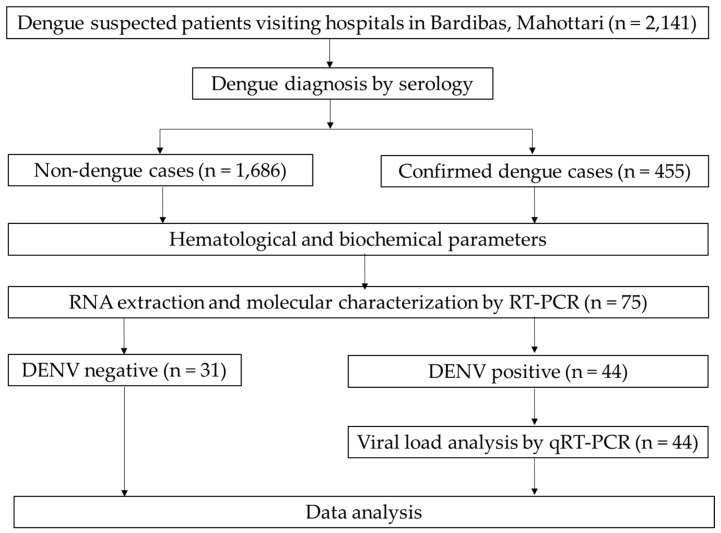
Flow diagram of the dengue study, Bardibas, Nepal, 2023.

Manual extraction of laboratory data was carried out from medical records and the laboratory data management system, and these were accessed using Excel 2019 (Figure 2).

### 2.4. Molecular Investigation

#### 2.4.1. Viral RNA Extraction and Quality and Quantity Assessment

QIAamp Viral RNA kit (QIAGEN, Hilden, Germany) was used for viral RNA extraction. Briefly, 140 μL of patient serum was subjected to lysis and elution as per the manufacturer’s manual. Viral RNA quantification and quality assessment were performed using NanoDrop (Thermo Fisher Scientific, Inc., Waltham, MA, USA).

#### 2.4.2. One-Step Reverse Transcription Polymerase Chain Reaction (RT-PCR)

The DENV RNA was detected by RT-PCR using a PrimeScriptTM One-Step RT-PCR Kit Ver. 2 (Takara Bio Inc., Shiga, Japan). Briefly, a final volume of 15 μL was used for the RT-PCR amplification, which contained 5 μL of template RNA. The RT-PCR reaction mixture contained 0.5 μL of enzyme mix, 6.5 μL of 2X buffer, 2 μL of nuclease-free water, and 0.5 μL of 100 pmol forward and reverse primers, with different primer sets (Table S1) for the detection of DENV and the identification of serotypes [15]. The RT-PCR program consisted of the following steps: 42 °C for 60 min; 35 cycles of 94 °C for 30 s, 55 °C for 1 min, 72 °C for 2 min, and this was carried out using a BioRad T100 Thermal Cycler (Bio-Rad Laboratories, Hercules, CA, USA). DNase-/RNase-free water (Sigma, New York, NY, USA) was used as a negative control, while a mixture of all four DENV serotypes (DENV-1 (99St12Astrain, PP422232), DENV-2 (00St22A, PP422234), DENV-3 (SLMC 50 strain, PP425980) and DENV-4 (SLMC 318 strain, PP422236)) with known viral concentrations (10^6^ FFU/mL of DENV-1, -3, and -4 and 10^7^ pfu/mL of DENV-2) was taken as a positive control [14]. Finally, the amplified PCR products were detected by agarose gel (1.5%) electrophoresis and observed in a gel documentation system (Azure Biosystems, Sierra Trinity, CA, USA).

#### 2.4.3. Quantification of DENV Genome Levels

A volume of 5 µL of total RNA extracted from serum was used for quantitative real-time RT-PCR (RT-qPCR) and amplification of the envelope gene was performed as described previously [26,27]. Briefly, the envelope gene was amplified by using a total of 20 µL of reaction mixture consisting of 5 μL of Taqman master mix, 9 µL of nuclease-free water, 0.3 µL of 100 pmol forward and reverse primers (DENV serotype-specific) and 0.4 µL of probe (Supplementary Table S1) of TaqMan Fast Virus 1-Step Master Mix (Life Technologies, Carlsbad, CA, USA). The RT-qPCR program consisted of the following steps: 50 °C for 5 min, 95 °C for 20 s, and 40 cycles at 95 °C for 3 s and 60 °C for 30 s, using the Quant Studio 5 real-time PCR system (Applied Biosystems, Foster City, CA, USA). The viral genome levels were expressed as genome copies/mL.

### 2.5. Data Analysis

Microsoft Excel was used for data entry. After proper cleaning and verification of data, SPSS version 25.0 for Windows (IBM Corp., Armonk, NY, USA) and GraphPad Prism version 9.3.0 for Windows (GraphPad Software, Boston, MA, USA) were used for statistical analysis. Continuous variables were presented as median (25–75% inter-quartile range (IQR)). The comparison of continuous variables between dengue vs. non-dengue, inpatients vs. outpatients, primary vs. secondary, and others was performed using the Mann–Whitney U test/median test and Kruskal–Wallis test as appropriate. All tests were considered statistically significant at an alpha error of 0.05. Maps were generated through ArcGIS Desktop version 10.8.2 (ESRI, Redlands, CA, USA).

## 3. Results

### 3.1. General Profiles of the Study Participants

**Figure 3:**
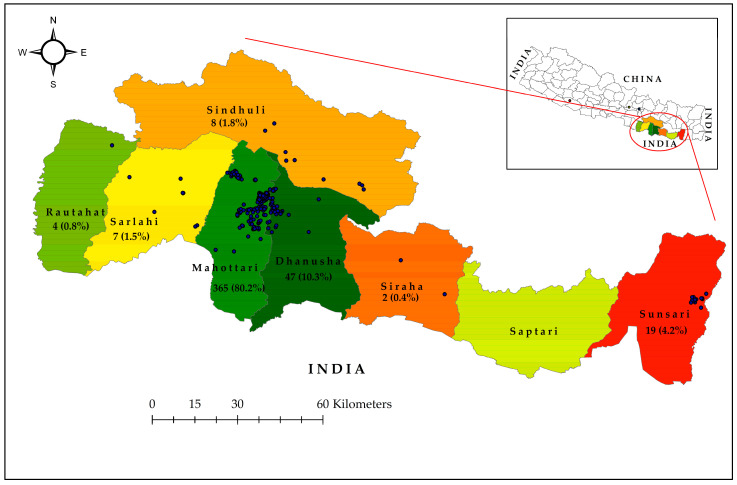
District-wise geographical mapping of laboratory-confirmed dengue cases enrolled in the study hospitals of Mahottari and surrounding areas in southern Nepal during the 2023 outbreak. Insert is a map of Nepal showing major districts of origin of confirmed dengue cases (color-coded), including single dengue case each from Dang, Dhading, and Kathmandu shown as dots (one dot = one case).

A total of 2141 dengue-suspected patients were investigated (age range: 1–94 years, median (IQR) age was 32 (20–47.5) years), with the majority (86.3%) being adults. Among them, 455 (21.3%) were dengue cases confirmed by serology and/or PCR, while the remaining 1686 (78.7%) were non-dengue patients. Among dengue-cases, 10.7% (44) were positive by both serological and molecular methods (Supplementary Table S2). Dengue infection was associated with gender [male = 1106 (51.7%) and female = 1035 (48.3%); *p* = 0.044], while the occurrence of DENV infections was higher among adults (413, (90.8%)) than children (42, (9.2%)). Further, the proportion of dengue in adults (413/1848, 22.3%) was significantly higher than in children (42/293, 14.3%) (*p* = 0.035). The vast majority of dengue cases were from Madhesh province (Mahottari, Dhanusha, Sarlahi, Rautahat, Siraha), comprising 425 cases (93.4%), with 19 (4.2%) from Koshi province (Sunsari) (Figure 3). The remaining 11 scattered cases (2.4%) were from Bagmati (Sindhuli, Kathmandu, and Dhading) and Lumbini (Dang) provinces.

### 3.2. Seasonal Trends of Dengue Cases in Bardibas, Mahottari

**Figure 4:**
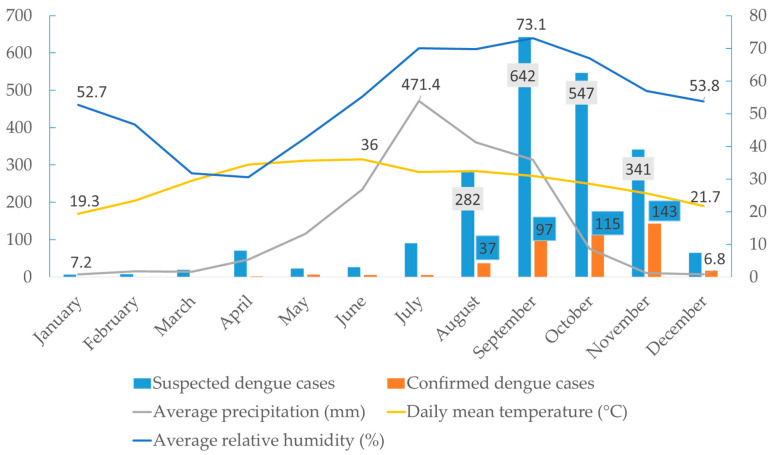
Seasonal trend of dengue cases and meteorological parameters reported in the study area during the 2023 outbreak in Nepal. In the figure, the left *Y*-axis denotes the number of suspected and confirmed dengue cases and average precipitation, whereas the right *Y*-axis denotes the daily mean temperature and average relative humidity.

Suspected dengue cases were reported in Bardibas from the beginning of the year, with a confirmed case in January. Following the onset of the monsoon season, increased relative humidity, along with favorable precipitation and temperature conditions for mosquito vectors, led to a rise in dengue cases starting in August (n = 37), with a peak in November (n = 143) (Figure 4). The four-month period from August to November accounted for the majority of cases (n = 392, 86.2%), reflecting a clear seasonal trend.

### 3.3. Blood Parameters of Dengue and Non-Dengue Patients

A significant difference was seen between dengue and non-dengue patients in the following laboratory parameters: hemoglobin, WBC, platelets, neutrophil, lymphocyte, eosinophil, monocyte, PCV, MCV, MCH, SGPT, and SGOT (*p* < 0.05) (Table 1). Particularly, WBC, platelet, and neutrophil counts were significantly lower, while liver enzymes (SGOT/SGPT), PCV, MCV, and MCH were significantly higher among dengue patients.

### 3.4. Blood Parameters of Dengue Patients by Admission Status

The dengue patients managed in the inpatient department (IPD) showed significantly lower hemoglobin than those in the outpatient department (OPD) (*p* = 0.005), while other parameters remained non-significant (*p* > 0.05) (Table 2). Male and female dengue patients significantly differed in terms of hemoglobin, RBC, PCV, and creatinine (*p* < 0.001) (Supplementary Table S3). Further, the MCHC value was found to be significantly higher in adults (*p* = 0.048) as compared to children (Supplementary Table S4).

### 3.5. Blood Parameters of Dengue Patients in Primary and Secondary Infection

The total bilirubin was significantly higher in secondary dengue infection compared to primary dengue infection (*p* = 0.032). Other blood parameters were not significantly different (*p* > 0.05), although lower platelets and higher ALP/SGOT were seen among secondary dengue patients (Table 3).

### 3.6. DENV Serotypes 1, 2, and 3 Detected During 2023 Dengue Outbreak in Southern Nepal

**Figure 5:**
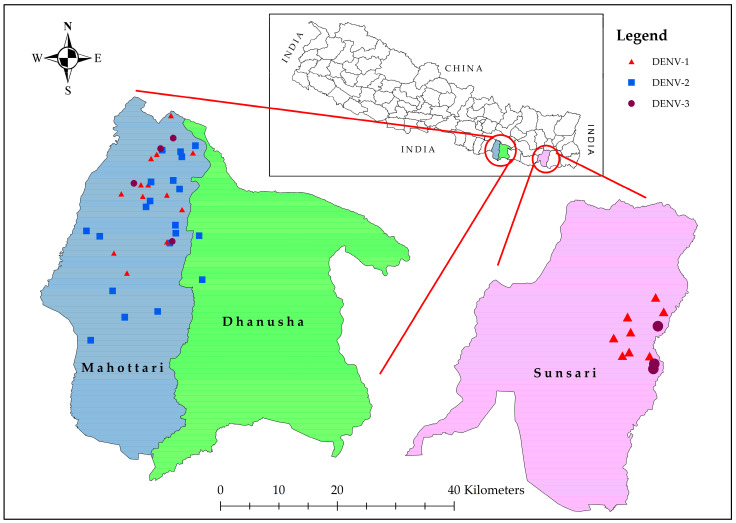
Distribution of DENV serotypes in southern Nepal during the 2023 outbreak.

A total of 75 serologically representative samples were analyzed for molecular detection, and among them, 44 (58.7%) were found to be DENV-positive by RT-PCR. During the 2023 outbreak in Mahottari, we identified the presence of DENV-1, -2, and -3 but not DENV-4 (Figure 5). Interestingly, DENV-1 and DENV-3 were introduced for the first in this area.

The dengue outbreak in Mahottari district in 2023 was predominantly caused by DENV-1 (n = 20) and DENV-2 (n = 18), together comprising 86.4% of the total dengue cases. DENV-3 cases were sporadic, making up six (13.6%) cases of the total. Additionally, there were five cases of co-infection with DENV-1 and DENV-2, representing about 11.4% of the cases (Figure 5).

In Bardibas municipality, all three serotypes were identified. Gaushala and Aurahi municipality in Mahottari district had dengue infection due to DENV-1 and -2. Similarly, only DENV-2 cases were confirmed in Mithila and Bateshwor municipality in Dhanusha district. Patients from Itahari (Sunsari district in Koshi province) had infection with DENV-1 and -3.

### 3.7. High Viral Load Detected in 2023 Dengue Outbreak in Mahottari and Surrounding Areas of Nepal

DENV-3 had a significantly higher median viral load (IQR) of 7.71 log_10_ copies/mL (6.48–7.94) compared to that of DENV-1 (6.72 log_10_ copies/mL [5.49–7.17]) and DENV-2 (4.76 log_10_ copies/mL [2.32–6.96]) (*p* = 0.036).

**Figure 6:**
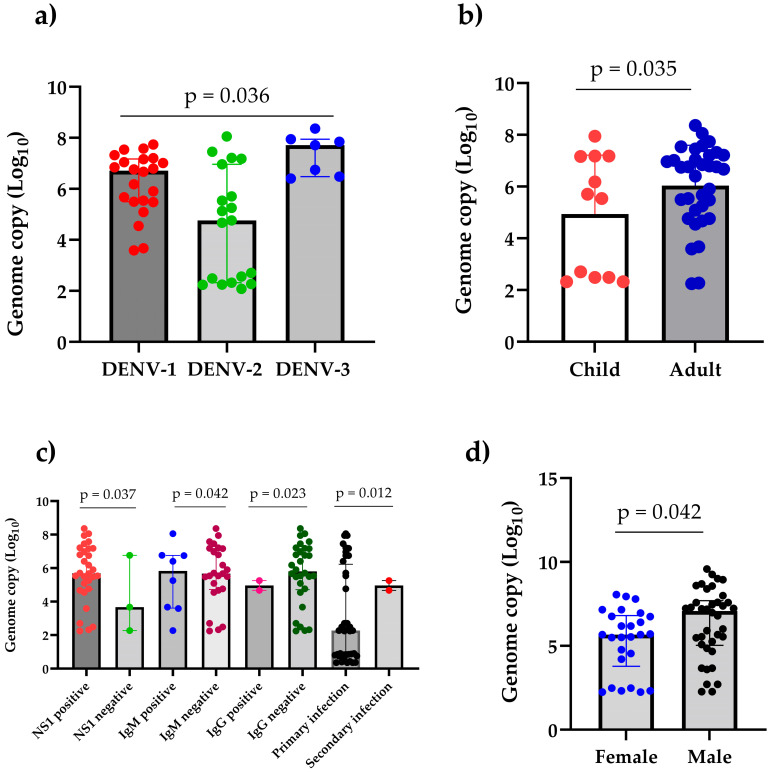
DENV viral load in 2023 dengue outbreak in Mahottari and surrounding areas of Nepal: (**a**) viral load by serotypes, (**b**) viral load by age groups, (**c**) viral load by sero-status, and (**d**) viral load by gender. The Mann–Whitney U test/median test was used to compare continuous variables between two groups as appropriate.

Adult patients exhibited significantly higher viral load [6.71 log_10_ copies/mL (5.02–7.21)] than children [5.63 log_10_ copies/mL (2.49–7.17)] (*p* = 0.035). There was significant difference in viral load by gender (*p* = 0.042), with male patients showing higher viral load [6.01 log_10_ copies/mL (4.76–6.96)] than female patients [5.51 log_10_ copies/mL (4.41–7.19)]. The viral load of NS1-positive patients [5.69 log_10_ copies/mL (4.74–7.16)] was significantly higher than NS1-negative patients [3.67 log_10_ copies/mL (2.27–6.76)] (*p* = 0.037). Similarly, DENV IgM-positive patients exhibited a significantly higher viral load [6.13 log_10_ copies/mL (3.61–6.76)] as compared to IgM-negative patients [5.67 Log_10_ copies/mL (4.72–7.17)] (*p* = 0.042). However, the viral load in IgG-negative patients was significantly higher [5.82 log_10_ copies/mL (4.72–7.16)] than in IgG-positive patients [5.05 log_10_ copies/mL (4.67–5.25)] (*p* = 0.023) (Figure 6). The viral load of patients with primary infection, with a median of 5.71 log_10_ copies/mL (4.54–7.16), was significantly higher than patients with secondary infection, with a median of 5.05 log_10_ copies/mL (4.67–5.25), (*p* = 0.012). Further, patients admitted to the emergency room exhibited a significantly higher viral load [7.15 log_10_ copies/mL (3.12–7.73)] compared to outpatients [5.69 log_10_ copies/mL (5.74–7.02)] (*p* = 0.037)] (Supplementary Table S5).

## 4. Discussion

We identified DENV-1 (45.5%) and DENV-2 (40.9%) as the major serotypes responsible for the 2023 dengue outbreak, with a small proportion of patients being infected by DENV-3 (13.6%) in Mahottari and surrounding districts of southern Nepal bordering India. This is the first report of the co-circulation of multiple (DENV-1, -2, and -3) serotypes in in this area, as only DENV-2 was reported previously from this region in 2017 [17]. This indicates the first introduction of DENV-1 and DENV-3 in these areas in 2023. The emergence of a new serotype or genotype may lead to the substitution of previously dominant strains; however, multiple serotypes may continue to co-circulate over time [28]. The sequential circulation of different DENV serotypes in a given area can be associated with a higher incidence of severe dengue cases in the future due to antibody-dependent enhancement (ADE) [29,30]. Due to the lack of systematic and genomic surveillance, it is not clear whether DENV-1 and -3 were introduced into this area in 2023 or whether they were there but remained unexplored due to no study being conducted. Therefore, this also highlights the need for continuous serotype/genomic surveillance to track the switching events on time, which would help in control and prevention and in informed policy-making.

The geographical distribution of dengue in Nepal is gradually changing and spreading to all districts of Nepal [15,19,31]. A serotype switching phenomenon has been observed during major outbreaks in Nepal in recent years. For instance, outbreaks were primarily driven by DENV-1 and DENV-2 in 2010, followed by a predominance of DENV-2 in 2013. DENV-1 re-emerged in 2016, with DENV-2 becoming dominant again in 2017. In 2019, both DENV-2 and DENV-3 circulated, while the 2022 outbreak had a co-circulation of DENV-1 and DENV-3 [15,16,17,32,33]. The serotype distribution of the Terai or hilly or mountain regions may differ, e.g., DENV-2 was the major contributor in the 2023 outbreak in Dhading (hilly region), central Nepal [15], and Jhapa, eastern Nepal [34], while both DENV-1 and DENV-2 were key players in our study area, i.e.,Mahottari (Terai region) in southern Nepal.

The observed gradual increase in dengue cases from June (n = 5) with a peak in November (n = 143) aligns closely with the seasonal patterns of temperature, relative humidity, and precipitation. The breeding, survival, and biting behavior of *Aedes* mosquitoes are directly affected by these environmental factors. In Nepal, the monsoon season typically spans from June to September, characterized by high rainfall and humidity, creating ideal conditions for mosquito proliferation due to the abundance of water sources for breeding [35]. The high number of dengue cases in November (post-monsoon) is likely due to the incubation period of the virus in both the vector and human host [36]. Similar seasonal trends have also been reported in other South Asian countries, where vector density and dengue incidence typically rise post-monsoon [37]. Further, the lack of proper waste management, inadequate public awareness, abundance of discarded tires with stagnant water, and poor sanitation facilities in this area create a favorable breeding environment for vectors. The influence of additional factors, such as urbanization, increased mobility, and higher adaptability of *Aedes* spp. on virus dissemination, cannot be ruled out despite the emergence of new serotypes. Bardibas, Mahottari, is a growing town and transit hub of national highways connecting Terai to Kathmandu, the capital city of Nepal [24]. Moreover, due to open borders, Bardibas receives a large influx of people from the neighboring dengue-endemic state of India (Bihar), which provides ample opportunity for DENV transmission [38]. Therefore, timely interventions of vector control measures before and during the monsoon season can help to mitigate the surge in dengue cases in the region.

We found a high viral load in patients infected with DENV-3 followed by DENV-1 and DENV-2. Generally, DENV-3 and -2 are responsible for severe disease with a high viral load [39]. A study in Singapore has demonstrated that DENV-1 often achieves a higher plasma viral load than DENV-2 [40]. Prior immunity can also modulate the replication and pathogenicity of subsequent serotype infections, thereby affecting disease severity and viral load [41]. However, it is not clear why this outbreak showed a high viral load despite most patients having primary infection. NS1-positive patients exhibited a significantly higher viral load compared to NS1-negative patients. This is true, as it is an indication of early acute infection that correlates well with a high viral load reflecting an active viral replication [42]. Moreover, NS1 also plays important role in pathogenesis/severity and enhanced viral replication [43]. The viral load of admitted and emergency patients was significantly higher than in outpatients. This is explained by the presence of warning signs and progressed severity in these patients to have a high viral load [39,44,45]. Like our findings, a previously published report also showed a significantly higher viral load in male patients than in female patients [46], perhaps due to hormonal influence. Adult patients had a significantly higher viral load compared to children, which is similar to a previous report from Latin America [47], despite the fact that children are the major victims of dengue worldwide. Nevertheless, the high viral load in patients increases the likelihood of transferring DENV to *Aedes* mosquitos [48,49], thereby affecting the disease transmission dynamics. However, additional investigations into serotype-specific variations in viral load dynamics may be required to enhance our understanding of DENV transmission in a specific population.

Bilirubin levels were significantly higher in patients with secondary dengue compared to primary, but the increment in liver enzymes did not reach statistical significance. This suggests an increased hepatic involvement in secondary dengue due to a more pronounced immune response being caused [50]. Interestingly, the blood parameters between inpatients and outpatients were not different. This might be because of public panic behaviors seen after dengue infection, which led to increased insistence on admission regardless of the severity levels. This is even more common in peripheral/community hospital settings like ours [15]. Increased public awareness and improving clinician compliance to national dengue management guidelines with adequate training will help in coping with situations like this.

In 2023, we confirmed more than 400 dengue cases in Bardibas alone, while the EDCD reported less than 100 cases [19]. This underscores a huge opportunity for improving dengue early warning and reporting systems and strengthening the national database. Efforts from the government/EDCD in combating dengue have notably enhanced over the past few years. However, existing gaps in the surveillance and reporting system should be addressed to enable more accurate disease burden estimation and facilitate evidence-based policy and strategy formulation. Dengue virus genomic surveillance is essential for monitoring the emergence and spread of different serotypes and genotypes, detecting mutations that may affect virus transmissibility or virulence, guiding vaccine development and effectiveness, and initiating timely public health interventions to control outbreaks and reduce disease burden.

Underreporting was likely due to both a lack of adequate communication between health authorities or inadequate reporting from local level and a lack of proper diagnostic facilities in peripheral health care settings. Thus, ensuring access to and availability of rapid dengue diagnostics in peripheral settings is one of the key areas requiring serious attention [51]. Furthermore, local governments should also establish a mechanism to ensure access to and availability of diagnostics and monitor for consistency and reliability of case reporting to the EDCD.

The vast majority were primary dengue infections. This means that the population was still naïve to DENV and more vulnerable to infection. Consequently, there is a high risk of a more severe epidemic with increased morbidity and mortality in the future. Understanding the distribution of DENV serotypes, its epidemiological patterns, laboratory parameters, and viral load kinetics in affected regions can support epidemic preparedness. This information is also crucial to anticipate possible complications and develop appropriate treatment strategies. We acknowledge the limitations—whole-genome sequencing of the DENV serotypes is currently underway, which will provide better insights into the virus’s origin, transmission, severity, and its implications for future vaccine development.

## 5. Conclusions

We concluded the co-circulation of DENV-1, -2, and -3 during the 2023 outbreak in Mahottari and neighboring areas of southern Nepal, with the first introduction of DENV-1 and -3 in this area. We found a high viral load in DENV-3 infection followed by DENV-1 and DENV-2. Viral load was higher among males and adults. Total bilirubin was significantly higher in secondary dengue than primary, suggesting an immune-mediated hepatic involvement. Dengue incidence in southern Nepal was found to be well correlated with seasonality, showing a typical rise in cases post-monsoon. Our findings suggest that dengue in this region has been largely underreported. This indicates that a national dengue surveillance system with enhanced reporting needs to be established with the involvement of local and federal governments to estimate the stratified burden of dengue in the country and initiate evidence-based intervention strategies. Therefore, the precise mapping of the DENV cases, mosquito vectors, and viral genomics through an integrated surveillance perspective are necessary for evidence-based policy-making to respond early and mitigate dengue-related mortality and morbidity in the country.

## Figures and Tables

**Table 1:** Blood parameters among dengue and non-dengue patients in hospitals of Bardibas, Mahottari, Nepal, 2023.

Blood Parameters	Total Number	Dengue Patient, Median (IQR)	Non-Dengue Patient, Median (IQR)	*p*-Value
Hemoglobin (g/dL)	1878	12.8 (11.6–14.2)	12.4 (11.3–13.7)	0.001
WBC (cells/µL)	1882	4000 (3000–5820)	7200 (5300–9502.5)	<0.001
RBC (million/µL)	1880	4.4 (4–4.8)	4.4 (4–4.8)	0.468
Platelet (cells/µL)	1880	131,000 (102,500–165,500)	175,000 (139,000–223,000)	<0.001
Neutrophil (%)	1872	63 (54.5–71)	70 (62–78)	<0.001
Lymphocyte (%)	1872	30 (23–39)	25 (17–31)	<0.001
Eosinophil (%)	1872	3 (2–3)	2 (2–3)	<0.001
Monocyte (%)	1872	3 (2–4)	3 (2–4)	<0.001
PCV (%)	1593	42.7 (37.5–48.4)	41.2 (36.1–46.55)	0.038
MCV (femtoliters)	1593	97 (87.75–106.5)	93.9 (86.1–102.8)	<0.001
MCH (pg)	1592	28.9 (26.6–30.6)	28 (26.1–30)	0.003
MCHC (g/dL)	1591	29.9 (27–32.6)	30.1 (27.3–32.6)	0.627
Bilirubin, total (mg/dL)	176	0.8 (0.7–0.9)	0.8 (0.7–0.9)	0.52
Bilirubin, direct (mg/dL)	176	0.2 (0.2–0.3)	0.2 (0.2–0.3)	0.54
ALP (U/L)	160	168.9 (130.5–205.1)	185.32 (118.2–234.8)	0.388
SGOT (U/L)	397	51 (34–88.1)	33.125 (25–50.6)	<0.001
SGPT (U/L)	401	40.3 (29.2–73.3)	28.6 (21.6–42.8)	<0.001
Total protein (g/dL)	168	6.5 (6.2–7)	6.7 (6.1–7)	0.652
Albumin (g/dL)	165	3.9 (3.8–4)	3.8 (3.7–4)	0.353
Creatinine (mg/dL)	584	0.895 (0.8–1)	0.86 (0.7–1)	0.187
Urea (mg/dL)	494	24.9 (20.5–30.4)	25.07 (20–30.8)	0.85
Sodium (mmol/L)	466	135.6 (134–138.3)	135.2 (134.2–136.9)	0.356
Potassium (mmol/L)	463	3.8 (3.7–3.9)	3.8 (3.7–4)	0.324

The Mann–Whitney U test was used to compare continuous variables between two groups. IQR, inter-quartile range; WBC, white blood cell; RBC, red blood cell; PCV, packed cell volume; MCV, mean corpuscular volume; MCH, mean corpuscular hemoglobin; MCHC, mean corpuscular hemoglobin concentration; SGPT, alanine aminotransferase; SGOT, aspartate aminotransferase; and ALP, alkaline phosphatase.

**Table 2:** Blood parameters of dengue patients by admission status in Bardibas, Mahottari, Nepal, 2023.

Blood Parameters	Total Number	Inpatients, Median (IQR)	Outpatients, Median (IQR)	*p*-Value
Hemoglobin (g/dL)	405	11.7 (9–12.3)	12.8 (11.7–14.2)	0.005
WBC (cells/µL)	405	3320 (2645–4360)	4000 (3010–5835)	0.196
RBC (million/µL)	405	4.0 (3.5–4.4)	4.4 (4–4.8)	0.081
Platelet (cells/µL)	403	110,000 (71,500–156,000)	131,000 (103,000–166,750)	0.604
Neutrophil (%)	403	54.5 (44.8–72.5)	64 (55–71)	0.420
Lymphocyte (%)	403	37.5 (21.5–50.0)	30 (23–39)	0.328
Eosinophil (%)	403	3 (2–3)	3 (2–3)	0.907
Monocyte (%)	403	3 (2.8–4.3)	3 (2–4)	0.665
PCV (%)	313	33.2 (28.6–43.4)	42.9 (37.7–48.4)	0.110
MCV (femtoliters)	313	90.5 (77.8–104.4)	97 (87.8–106.6)	0.788
MCH (pg)	313	25.7 (22.1–28.1)	28.9 (26.8–30.6)	0.130
MCHC (g/dL)	312	30.1 (22.5–34.4)	29.9 (27.1–32.6)	0.764
Bilirubin, total (mg/dL)	26	0.73 (0.6–0.8)	0.8 (0.7–1.0)	1.000
Bilirubin, direct (mg/dL)	26	0.205 (0.18–0.23)	0.2 (0.2–0.3)	0.483
ALP (U/L)	25	161.3 (148.4–174.2)	168.9 (125.8–208.5)	1.000
SGOT (U/L)	65	67.7 (28.5–216.0)	49.6 (34.0–88.0)	0.584
SGPT (U/L)	65	34.9 (13.5–63.1)	41.9 (29.3–78.2)	0.628
Total protein (g/dL)	25	6.6 (6.2–6.9)	6.5 (6.2–7.0)	1.000
Albumin (g/dL)	25	3.7 (3.6–3.9)	3.9 (3.8–4.0)	0.520
Creatinine (mg/dL)	107	0.9 (0.9–1.0)	0.9 (0.8–1.0)	1.000
Urea (mg/dL)	84	28.4 (23.6–31.4)	24.6 (20.4–30.4)	0.409
Sodium (mmol/L)	81	134.6 (133.0–147.9)	135.7 (134.0–138.3)	0.766
Potassium (mmol/L)	81	3.5 (3.2–3.8)	3.8 (3.7–3.9)	0.453

The Mann–Whitney U test was used to compare continuous variables between two groups. IQR, inter-quartile range; WBC, white blood cell; RBC, red blood cell; PCV, packed cell volume; MCV, mean corpuscular volume; MCH, mean corpuscular hemoglobin; MCHC, mean corpuscular hemoglobin concentration; SGPT, alanine aminotransferase; SGOT, aspartate aminotransferase; and ALP, alkaline phosphatase.

**Table 3:** Blood parameters of dengue patients in primary and secondary infection, Mahottari, Nepal, 2023.

Blood Parameters	Total Number	Primary, Median (IQR)	Secondary, Median (IQR)	*p*-Value
Hemoglobin (g/dL)	405	12.8 (11.7–14.2)	11.65 (10.55–13.3)	0.164
WBC (cells/µL)	405	4000 (3000–5800)	3880 (2567.5–6327.5)	0.881
RBC (million/µL)	405	4.4 (4–4.8)	4.2 (3.7–4.8)	0.34
Platelet (cells/µL)	403	132,000 (103,000–166,000)	120,000 (89,750–160,000)	0.276
Neutrophil (%)	403	63 (55–71)	65.5 (54–72)	0.81
Lymphocyte (%)	403	30 (23–39)	29.5 (23.3–37.5)	0.621
Eosinophil (%)	403	3 (2–3)	3 (2–3.8)	0.476
Monocyte (%)	403	3 (2–4)	3.5 (2.3–4)	0.351
PCV (%)	313	42.8 (37.6–48.6)	41 (35–47.8)	0.99
MCV (femtoliters)	313	96.9 (87.7–105.8)	104 (89.1–113.1)	0.952
MCH (pg)	313	28.9 (26.7–30.6)	28.7 (23.6–30.3)	0.646
MCHC (g/dL)	312	30 (27.1–32.6)	28.4 (22.7–31.05)	0.587
Bilirubin, total (mg/dL)	26	0.8 (0.7–0.8)	1.1 (1–1.9)	0.032
Bilirubin, direct (mg/dL)	26	0.23 (0.2–0.3)	0.4 (0.3–0.4)	0.085
ALP (U/L)	25	168.0 (124.4–203.4)	188.6 (137.9–202.6)	0.593
SGOT (U/L)	65	50.3 (33.1–93.9)	52 (44.4–98.6)	0.978
SGPT (U/L)	65	41.1 (29.2–77.1)	32.5 (29–76)	0.356
Total protein (g/dL)	25	6.66 (6.2–7.0)	6.2 (6–6.4)	0.48
Albumin (g/dL)	25	3.9 (3.8–4)	3.6 (3.6–3.6)	0.52
Creatinine (mg/dL)	107	0.9 (0.8–1.0)	0.8 (0.6–1.2)	0.674
Urea (mg/dL)	84	24.9 (20.52–30.1)	27.3 (14.4–34.1)	0.694
Sodium (mmol/L)	81	135.5 (134–137.9)	137.6 (135.3–138.8)	0.181
Potassium (mmol/L)	81	3.8 (3.7–3.9)	3.7 (3.6–4.0)	0.915

The Mann–Whitney U test was used to compare continuous variables between two groups. IQR, inter-quartile range; WBC, white blood cell; HCT, hematocrit; RBC, red blood cell; MCV, mean corpuscular volume; MCH, mean corpuscular hemoglobin; MCHC, mean corpuscular hemoglobin concentration; SGPT, alanine aminotransferase; SGOT, aspartate aminotransferase; and ALP, alkaline phosphatase.

## Data Availability

The datasets generated and/or analyzed during the current study are available in the manuscript and the Supplementary Materials.

## Supplementary Materials

The following supporting information can be downloaded at: https://www.mdpi.com/article/10.3390/pathogens14070639/s1, Table S1: Primers and probes for Conventional and Real time RT-PCR. Table S2: Dengue positive patients by both serological and molecular methods. Table S3: Blood parameters of male and female dengue patients, Mahottari, Nepal, 2023. Table S4: Blood parameter profiles of children and adults, Mahottari, Nepal, 2023. Table S5: Viral load in different category, Mahottari, Nepal, 2023.
