# Supplementary material for: Emergence of Dengue Virus Serotypes 1 and 3 in Mahottari and Adjacent Areas of Southern Nepal
Source: Pathogens. 2025 Jun 26;14(7):639. doi: 10.3390/pathogens14070639 (PMC12299309; doi:10.3390/pathogens14070639)
Supplement: Supplementary file 1 [file pathogens-14-00639-s001.zip › pathogens-3628630-supplementary.pdf]

Supplementary data

Supplementary Table S1. Primers and probes for Conventional and Real time RT-PCR

Assay	Identification	Sequences (5' - 3')	Size (base pair)
**Conventional RT-PCR**	Dengue Consensus primer		
	DC-1, Forward	TCAATATGCTGAAACGCGCGAGAAACCG	
	DC-2, Reverse	TTGCACCAACAGTCAATGTCTTCAGGTTC	511
	Dengue serotype primer		
	D1-Forward	GGACTGCGTATGGAGTTTTG	
	D1-Reverse	ATGGGTTGTGGCCTAATCAT	490
	D2-Forward	GTTCCTCTGCAAACACTCCA	
	D2-Reverse	GTGTTATTTTGATTTCCTTG	230
	D3-Forward	GTGCTTACACAGCCCTATTT	
	D3-Reverse	CCATTCTCCCAAGCGCCTG	320
	D4-Forward	CCATTATGGCTGTGTTGTTT	
	D4-Reverse	CTTCATCCTGCTTCACTTCT	399
**Dengue serotype specific Real time RT-PCR**	D1-469 Forward	GAACATGGRACAAYTGCAACYAT	67
	D1-536 Reverse	CCGTAGTCDGTCAGCTGTATTTCA	
	MGB-493 probe	ACACCTCAAGCTCC	
	D2-493 Forward	ACACCACAGAGTTCCATCACAGA	68
	D2-568 Reverse	CATCTCATTGAAGTCNAGGCC	
	MGB-545 probe	CGATGGARTGCTCTC	
	D3-1 Forward	ATGAGATGYGTGGGAGTRGGAAAC	70
	D3-71 Reverse	CACCACDTCAACCCACGTAGCT	
	MGB-27 probe	AGATTTTGTGGAAGGYCT	
	D4-711 Forward	GGTGACRTTYAARGTHCCTCAT	75
	D4-786 Reverse	WGARTGCATRGCTCCYTCCTG	
	TAMPRA-734 probe	CCAAGAGACAGGATGTGACAGTGCTRGGATC	

Supplementary Table S2. Dengue positive patients by both serological and molecular methods

Dengue status	Positive	Negative	Total
Serological method	51	24	75
Molecular method	44	31	75

Supplementary Table S3. Blood parameters of male and female dengue patients, Mahottari, Nepal, 2023

**Blood parameters**	**Male, Median (IQR)**	**Female, Median (IQR)**	**P-value**
Hemoglobin (g/dL)	13.8 (12.5-14.9)	11.95 (11-12.8)	**<0.001**
WBC (cells/μL)	4130 (3200-5950)	3845 (2800-5592.5)	0.11
RBC (million/μL)	4.6 (4.24-5)	4.1 (3.815-4.5)	**<0.001**
Platelet (cells/μL)	129000 (94000-161250)	132000 (112000-171000)	0.67
Neutrophil (%)	64 (56-71)	63 (54-72)	0.96
Lymphocyte (%)	30 (23-38)	31 (23-40)	0.28
Eosinophil (%)	3 (2-3)	3 (2-3)	0.43
Monocyte (%)	3 (2-4)	3 (2-4)	0.15
PCV (%)	44.75 (38.975-50.4)	41.1 (33.9-45.5)	**<0.001**
MCV (femtoliters)	96.85 (88.95-107.25)	97 (86.4-105.8)	0.97
MCH (pg)	29.25 (27.375-30.8)	28 (25.1-29.9)	0.01
MCHC (g/dL)	30.25 (27.9-32.65)	29.6 (26-32.525)	0.49
Bilirubin- Total (mg/dL)	0.8 (0.755-0.965)	0.78 (0.64-0.82)	0.42
Bilirubin- Direct (mg/dL)	0.23 (0.2-0.305)	0.23 (0.205-0.27)	1.00
ALP (U/L)	168.9 (123-200.05)	168.1 (138.5-258.3)	1.00
SGOT (U/L)	53.585 (38.65-86.28)	49.59 (28-142.69)	0.69

SGPT **(**U/L**)**			
	40.3 (31.4-77.0625)	40.33 (21.3-70.7)	0.92
Total protein **(**g/dL**)**			
	6.45 (6.0575-6.8)	6.9 (6.22-7.105)	0.23
Albumin **(**g/dL**)**			
	3.895 (3.63-4)	3.9 (3.8-4.015)	1.00
Creatinine **(**mg/dL**)**			
	1 (0.9-1.12)	0.84 (0.705-0.895)	**<0.001**
Urea **(**mg/dL**)**			
	28.27 (22.135-31.65)	23.5 (19.05-26.95)	0.06
Sodium **(**mmol/L**)**			
	135.005 (134-137.65)	135.85 (134.05-138.83)	0.66
Potassium **(**mmol/L**)**			
	3.805 (3.7-3.9)	3.795 (3.52-3.9)	0.37

The Mann–Whitney U test and independent samples t-test was used to compare continuous variables between two groups. IQR, inter-quartile range; WBC, white blood cells; RBC, red blood cells; PCV, packed cell volume; MCV, mean corpuscular volume; MCH, mean corpuscular hemoglobin; MCHC, mean corpuscular hemoglobin concentration; SGPT, alanine aminotransferase; SGOT, aspartate aminotransferase; and ALP, alkaline phosphatase.

Supplementary Table S4: Blood parameter profiles of children and adults, Mahottari , Nepal, 2023

Blood parameters	Child, Median (IQR)	Adult, Median (IQR)	P-value
Hemoglobin (g/dL)	12.8 (11.6-13.85)	12.8 (11.6-14.2)	0.991
WBC (cells/μL)	4230 (3300-7360)	4000 (3000-5565)	0.907
RBC (million/μL)	4.42 (4.055-4.71)	4.4 (3.9375-4.8125)	0.775
Platelet (cells/μL)	142000 (117000-195000)	130000 (99250-164000)	0.283
Neutrophil (%)	62 (52.25-69.25)	64 (55-71.5)	0.629
Lymphocyte (%)	30 (26.25-38.75)	30 (22-39)	0.828
Eosinophil (%)	3 (2-3.75)	3 (2-3)	0.208
Monocyte (%)	3 (2-4)	3 (2-4)	0.237
PCV (%)	42.5 (40.3-48.6)	42.75 (37.175-48.4)	0.986
MCV (femtoliters)	100 (94.2-105.8)	96.3 (87.4-106.6)	0.195
MCH (pg)	27.9 (25.1-29.2)	29 (26.8-30.6)	0.063
MCHC (g/dL)	28.2 (26.1-30.2)	30.2 (27.05-33)	**0.048**
Creatinine (mg/dL)	0.7 (0.51-0.84)	0.91 (0.8-1.015)	0.36
Urea (mg/dL)	20.9 (16.45-21.85)	26.1 (20.64-30.535)	0.069
Sodium (mmol/L)	135 (134-137.4)	135.6 (134-138.325)	0.496
Potassium (mmol/L)	3.845 (3.84-3.93)	3.8 (3.6875-3.9)	0.367

The Mann–Whitney U test and independent samples t-test was used to compare continuous variables between two groups. IQR, inter-quartile range; WBC, white blood cells; RBC, red blood cells; PCV, packed cell volume; MCV, mean corpuscular volume; MCH, mean corpuscular hemoglobin; MCHC, mean corpuscular hemoglobin concentration; SGPT, alanine aminotransferase; SGOT, aspartate aminotransferase; and ALP, alkaline phosphatase.

Supplementary Table S5: Viral load in different category, Mahottari, Nepal, 2023

Particulars	Category	Viral load Log_{10} (copies per ml)	P-value
Dengue serotypes	DENV-1	6.72 (5.49-7.17)	0.036
	DENV-2	4.76 (2.32-6.96)	
	DENV-3	7.71 (6.48-7.94)	
Gender	Male	6.01 (4.76-6.96)	0.042
	Female	5.51 (4.41-7.19)	
Age group	Child	5.63 (2.49-7.17)	0.035
	Adult	6.71 (5.02-7.21)	
Sero status	NS1 positive	5.69 (4.74-7.16)	0.037
	NS1 negative	3.67 (2.27-6.76)	
	IgM positive	6.13 (3.61-6.76)	0.042
	IgM negative	5.67 (4.72-7.17)	
	IgG positive	5.82 (4.72-7.16)	0.023
	IgG negative	5.05 (4.67-5.25)	
Immune status	Primary	5.705 (4.54-7.16)	0.012
	Secondary	5.05 (4.67-5.25)	
Patients type	Emergency patients	7.15 (3.12-7.73)	0.037
	Outpatients	5.69 (5.74-7.02	

The Mann–Whitney U test/Median text was used to compare continuous variables between two groups as appropriate
